# Correction to: 11 Abstracts from UL Hospitals NCHD Conference

**DOI:** 10.1007/s11845-025-03959-8

**Published:** 2025-05-06

**Correction to: Irish Journal of Medical Science (1971 -)**

10.1007/s11845-025-03911-w

This abstracts was initially published as non-open access despite being intended for open access publication. This issue has now been resolved.

The original article has been corrected.

